# Factors associated with severity and anatomical distribution of diabetic foot ulcer in Uganda: a multicenter cross-sectional study

**DOI:** 10.1186/s12889-023-15383-7

**Published:** 2023-03-10

**Authors:** Bienfait Mumbere Vahwere, Robinson Ssebuufu, Alice Namatovu, Patrick Kyamanywa, Ibrahim Ntulume, Isaac Mugwano, Theophilus Pius, Franck Katembo Sikakulya, Okedi Francis Xaviour, Yusuf Mulumba, Soria Jorge, Gidio Agaba, George William Nasinyama

**Affiliations:** 1 Kampala International University, Western Campus, Kampala, Uganda; 2 Mengo Hospital, Kampala, Uganda; 3 College of Veterinary Medicine, Animal Resources and Biosecurity, Makerere University, Kampala, Uganda; 4 Uganda Martyrs University, Nkozi, Uganda; 5 Fortportal Regional Referral Hospital, Fort portal, Uganda; 6 Faculty of Medicine, Université Catholique du Graben, Butembo, Democratic Republic of the Congo; 7 Uganda Cancer Institute, Kampala, Uganda; 8 Kiruddu National Referral Hospital, Kampala, Uganda; 9 Unicaf University in Uganda, Kampala, Uganda

**Keywords:** Diabetic foot ulcer, Severity, Uganda

## Abstract

**Background:**

Diabetic foot ulcer (DFU) is a devastating complication of diabetes mellitus (DM) that is associated with increased mortality, morbidity, amputation rate and economic burden. This study aimed at identifying the anatomical distribution and factors associated with severity of DFU in Uganda.

**Methodology:**

This was a multicenter cross-sectional study conducted in seven selected referral hospitals in Uganda. A total of 117 patients with DFU were enrolled in this study between November 2021 and January 2022. Descriptive analysis and modified Poisson regression analysis were performed at 95% confidence interval; factors with p-value < 0.2 at bivariate analysis were considered for multivariate analysis.

**Results:**

The right foot was affected in 47.9% (n = 56) of patients, 44.4% (n = 52) had the DFU on the plantar region of the foot and 47.9% (n = 56) had an ulcer of > 5 cm in diameter. The majority (50.4%, n = 59) of patients had one ulcer. 59.8% (n = 69) had severe DFU, 61.5% (n = 72) were female and 76.9% had uncontrolled blood sugar. The mean age in years was 57.5 (standard deviation 15.2 years). Primary (p = 0.011) and secondary (p < 0.001) school educational levels, moderate (p = 0.003) and severe visual loss (p = 0.011), 2 ulcers on one foot (p = 0.011), and eating vegetables regularly were protective against developing severe DFU (p = 0.03). Severity of DFU was 3.4 and 2.7 times more prevalent in patients with mild and moderate neuropathies (p < 0.01), respectively. Also, severity was 1.5 and 2.5 higher in patients with DFU of 5–10 cm (p = 0.047) and in those with > 10 cm diameter (p = 0.002), respectively.

**Conclusion:**

Most DFU were located on the right foot and on the plantar region of the foot. The anatomical location was not associated with DFU severity. Neuropathies and ulcers of > 5 cm diameter were associated with severe DFU but primary and secondary school education level and eating vegetables were protective. Early management of the precipitating factors is important to reduce the burden of DFU.

## Background

Diabetic Foot Ulcer (DFU) is an advanced consequence of diabetes mellitus (DM). There is a 15% lifetime chance of developing the ulcer among diabetic patients and when it occurs, it is associated with high mortality [1]. The 5-year survival rate among patients with DFU varies between 25% and 45% worldwide [2–4]. Globally, DFU has become one of the leading causes of lower limb amputation with over 1 million patients amputated annually, an average of a limb amputation every 20 s [5]. Following amputation for DFU, 85% of patients will still develop chronic infection and other forms of gangrene which lead to poor quality of life and financial stress [6, 7]. One third of the management costs of diabetes is estimated to be linked to foot ulcers as compared to patients without DFU and the cost of care is estimated to be 5.4 and 2.6 times higher in the year of first episode and second episode, respectively [8]. The cost of treatment also increases with severity of the DFU with the highest grade DFU ulcers costing eight times higher than the lowest grade [7]. In addition to high management costs, the severity of DFU ultimately leads to high mortality [9]. The prevalence of diabetic foot has been reported to be higher among people with type 2 DM compared to those with type 1 diabetes worldwide [10]. Globally, the prevalence of DFU averages at 6.4% with a higher predilection in men compared to women [11]. This prevalence varies between 3% in Oceania to 13% in North America, with a prevalence of 7.2% across Africa; in Uganda it varies from 1 to 4% [10]. In Ethiopia, a prevalence of 13.6% of DFU was reported among type 2 diabetes mellitus patients and it was associated with rural residence, poor foot self-care practice, obesity, and neuropathy [12].

Studies have shown that factors usually associated with occurrence of DFU include older age, longer duration of DM, hypertension, diabetic retinopathy and smoking history [13]. Peripheral neuropathy, peripheral vascular disease and foot trauma were also reported risk factors in the pathophysiology of foot ulcer [14]. Other factors include low educational status, high body mass index (BMI)and inadequate foot self-care practice [15–17], however these factors may differ based on the patient’s socio-economic status, demographic characteristics, and the evolution of DFU within the facility [12]. The factors precipitating progression to severe DFU in Ugandan patients have not been well studied.

The management of DFU requires the participation of the patient [18] and the outcome is related to patient awareness and self-foot care behavior [19–21]. Self-management of DM has a significant impact on the outcome of blood sugar level control and complications such as DFU [18].

Although Wagner grade 3 and 4, metatarsal is the most common location of DFU and associated factors have been largely studied in developed countries [19–23], there is still a paucity of data regarding the anatomical distribution and factors associated with severe DFU among patients in LMICs such as Uganda. An evidence-based understanding of the factors associated with increasing severity of DFU is necessary to establish effective control measures to reduce its burden. The purpose of this study, therefore, was to determine the anatomical distribution and factors associated with increasing severity of DFU in patients with DM in Uganda.

## Methods

### Study design and setting

**Fig. 1:**
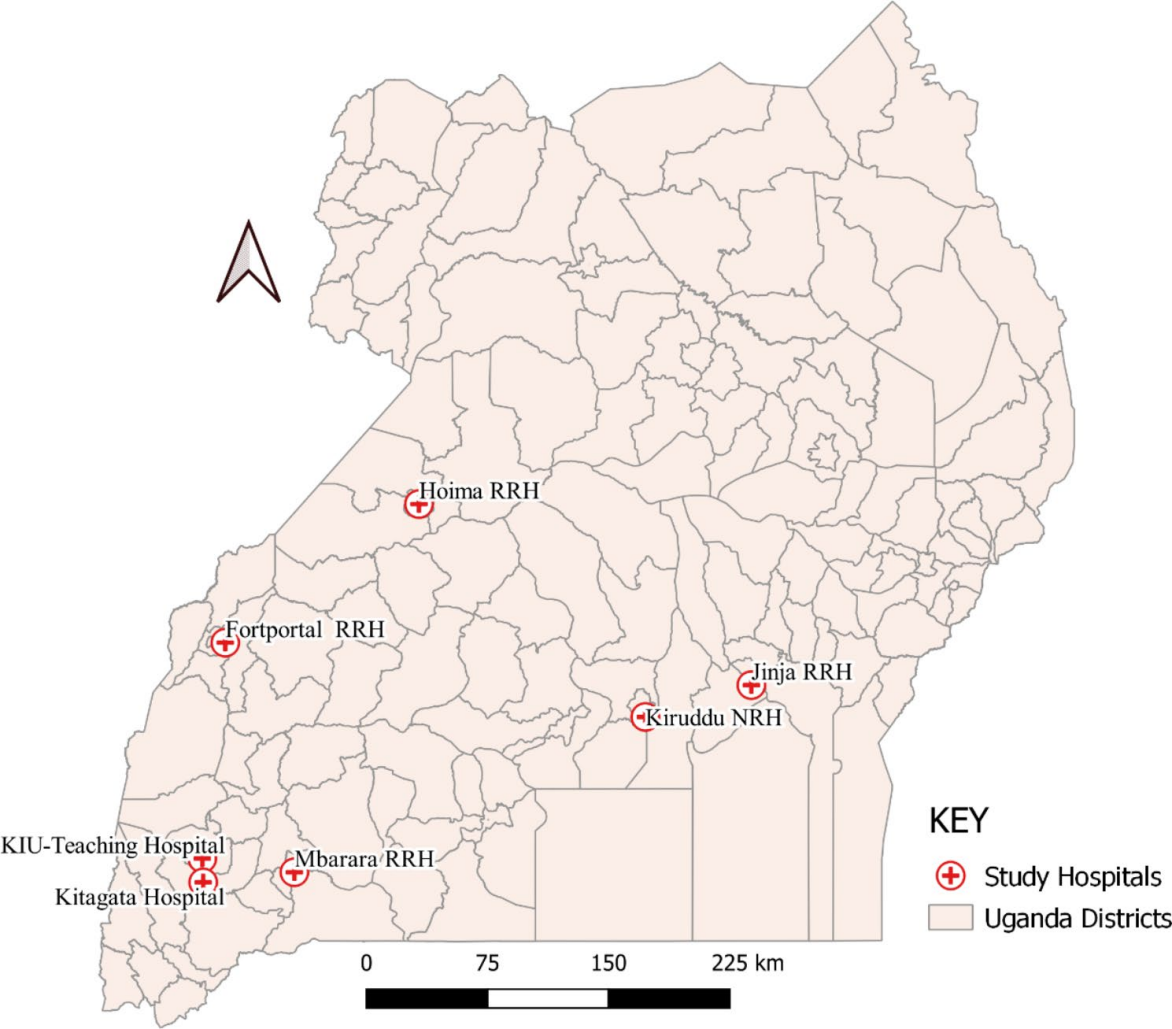
Distribution of study sites according to district. RRH: Regional referral hospital; NRH: National referral hospital

This multicenter cross-sectional study was conducted in 7 hospitals in Uganda (Kampala International Teaching Hospital, Kitagata General Hospital, Mbarara Regional Referral Hospital, Fort Portal Regional Referral Hospital, Hoima Regional Referral Hospital, Jinja Regional Referral Hospital and Kiruddu Specialized Hospital) (Fig. 1). The hospitals were deliberately chosen based on the high prevalence of DM in Central, Western and Eastern Uganda where the hospitals are located [24].

### Patients and recruitment

All patients aged 18 years and above with DM type 1 and type 2, having a wound located below the ankle and attending the surgical department and/or DM clinics of selected hospitals in Uganda between November 1, 2021 and January 31, 2022 were recruited. Purposive.

consecutive sampling method was used until the desired sample size was reached. Patients provided written informed consent to participate in the study. Patients without mental capacity and those without an adult to consent for them were excluded from the study.

DFU patients with communication difficulty, such as those with severe cognitive impairment or those who could not consent, were excluded from the study.

The required sample size for the study patients with DFU was calculated using the Kish Leslie formula as cited by Singh [25]. Data about the prevalence of DFU in Uganda is still scanty, therefore we used the prevalence estimate of DFU in a cross-sectional study done in Egypt (8.7% among adult patients aged 18 years and above attending Alexandria University Teaching Hospital Diabetic clinic) to determine the sample size [26]. Using the prevalence estimate from Egypt, which is similar to the one in Kenya [27], resulted in a calculated sample size of 122 patients.

### Study procedure

We developed and piloted a data collection tool informed by relevant literature. The tool was translated by a language expert into the local languages spoken in each of the study areas, namely Luganda/Lusoga, Runyakore/Rukiga and Runyoro/Rutoro. A medical doctor, an ophthalmologist/ophthalmology clinical officer, a nurse and a surgical resident were recruited and trained as research assistants in each of the selected hospitals. Data collection was supervised by the principal investigator (BMV) and other authors (RS, GA, IM, and FKS).

Physical examination to assess the Wagner classification, anatomical distribution of DFU, neuropathy, and blood pressure was performed by a doctor. The ankle-brachial index (ABI) was calculated using the ratio of the blood pressure taken at the ankle of the affected foot and the blood pressure taken at the one third distal of the arm. The ABI was not measured for patients with DFU affecting the ankle and the variable was considered as “not applicable”. The key variables included socio-demographic variables such as age, sex, occupation, monthly income, tribe and residency, general characteristics of the patients such as type of DM, duration of DM, history of trauma, duration of the diabetic foot ulcer, type of therapy and behavior-related factors such as history of smoking, alcohol intake, type of diet, frequency of consulting the diabetic clinic, foot care and lifestyle. Patients were asked about their regular weekly diet and the food was categorized depending on the main constituent: carbohydrates (matoke/plantains, cassava, posho, rice, sweet potatoes, Irish potatoes, pumpkins, yams), proteins (meat, eggs, fish, beans, milk), lipids (ground nuts, sim sim paste, ghee, cooking oil) and fruits and vegetables. Frequency of consumption was categorized as: daily (if they ate the mentioned food in a category every day), usually (if they ate any of the foods in the category at least 4 to 6 times in a week), occasionally (if at least 2 to 3 times in a week) and rarely (if once a week or less frequent). Regarding alcohol intake, the patient was regarded as an alcoholic if they drink alcohol regularly irrespective of the type of alcohol and non-alcoholic if the patient does not take alcohol at all, if the patient drinks alcohol once in a while or if a patient had completely stopped drinking alcohol for two years.

For the smoking variable, a smoker was a patient who chewed or smoked tobacco irrespective of the type and number of times per day whilst a non-smoker status referred to a patient who has never smoked or one that has ceased smoking for at least two years. Diabetic clinic consultation was assessed as the frequency of consultation and as categorized as monthly, every 6 months, once in a year or not at all. Foot care of the patients specifically considered what the patient uses to cut the nails (nail cutter, knife, or razor blade),who cuts the nails for the patient (self or assisted by another person) and the type of shoes regularly worn by the patient (closed, open or fitted) while the foot score consisted of: washing feet - score 1; drying in between the toes - score 2; and using moisturizing products - score 3. Based on this scoring, a good score meant a patient practices all three, a medium score if patient practices any two and a poor score if the patient practices only one of the three.

Blood samples for HbA1c and fasting blood sugar (FBS) testing were collected from one of the main superficial veins of the cubital fossa (cephalic, basilic, median cubital, and median ante brachial**).**The blood sample was collected in an ethylenediaminetetraacetic acid (EDTA) grey top vacutainer for blood sugar tests and glycosylated hemoglobin (HbA1c) [22, 28] and transported in a cooler box (AL medical cool box, England UK) at 2 to 6 Celsius degree to the laboratory of Kampala International University-Teaching Hospital (KIU-TH) [29]. Four milliliters (ml) of blood were withdrawn from the anterior cubital fossa of each subject using a sterile disposable syringe and needle after cleaning the site with a swab soaked in 70% alcohol.

HbA1c was analyzed using aIchroma II Machine (2017) and results were interpreted as follows: <6.5% HbA1c was considered as controlled DM and an HbA1c of 6.5% and above were uncontrolled DM [30]. Each study patient received a printed copy of their results.

Patients’ weight in kilograms and height in meters were determined using a calibrated analogue weighing scale and wall mounted station meter manufactured by Southern Early Child Association (SECA). The body mass index was calculated using the formula: BMI = kg/m^2^ where kg was a patient’s weight in kilograms and m^2^ was their height in meters squared. Patients were categorized as normal (BMI of 18.5 to 24.9), overweight (BMI of 25.0 to 29.9) or obese (BMI of 30 and above) [31]. Blood pressure was taken using a digital automated sphygmomanometer (bosomedicus vital D-72,417 JUNGINGEN/ German) with an appropriate cuff size for the arm. High blood pressure was defined as systolic blood pressure ≥ l40 mmHg or diastolic pressure ≥ 90 mmHg (European Society of Cardiology/European Society of Hypertension, 2018) [32].

Pressure sensation was assessed using 5.07/10 g monofilament (Semmes Weinstein monofilament test, made in China) at 4 of the 10 standard sites of the sole of the feet (plantar base of the big toe, 2nd and 5th toes and at the heel), avoiding areas with callosity [33]. Vibration sense was elicited using a 128 Hz tuning fork at the hallux [33, 34]. Diabetic neuropathy was classified as absent, minor, moderate or severe using the new classification by Picon and collaborators [35]. The Neuropathy Disability Score (NDS) was used to assess the grade of diabetic peripheral neuropathy (DPN) for each patient. The NDS system is a neuropathy scoring tool ranging from 0 to 10 which can also be used for assessment of severity of peripheral neuropathy by considering four parameters: vibration sense by using a 128 Hz tuning fork (0 = present,1 = reduced/absent for each foot), temperature sensation by using a cold tuning fork (0 = present,1 = reduced/absent for each foot), pin-prick sensation by a monofilament test (0 = present,1 = reduced/absent for each foot and ankle reflex), Achilles tendon reflex by using a patellar hammer (0 = normal, 1 = present with reinforcement, 2 = absent per side) [36]. Absence of neuropathy (normal) was considered when the score was 0 to 2. The classification of DPN disability was graded as follows: mild (score 3–5), moderate (score 6–8), and severe (score 9–10) as described by Dyck [37].

Patients’ feet were examined to determine the characteristics of the foot ulcer, number of ulcers, size of ulcers and location. The ulcer was classified using the Wagner classification [23].

The patients were classified into two (2) categories based on severity of DFU, as early DFU (less severe) (grade 1 and grade 2 foot ulcer) and severe or late DFU (grade 3 and above) using the Wagner classification [9].

Visual loss was assessed by either a trained ophthalmology clinical officer or ophthalmologist using a Snellen chart to assess the smallest letter a patient can read or orientation of letters at 20 feet or 6 m with one eye closed. Visual impairment was categorized according to the WHO classification where low vision is classified into three categories: mild, moderate, and severe. Mild visual impairment is visual acuity of ≥ 6/18, moderate visual impairment is less than 6/18 but equal or better than 6/60, whilst severe visual impairment is < 6/60 [38].

### Data processing and analysis

The raw data was entered into MS Excel spreadsheet software, cleaned and later exported to Stata version 15 (Stata Corp®) for analysis. Categorical variables were analyzed using the proportions whilst the difference in proportions was assessed using the Chi-square test and data was summarized and presented in form of frequencies and percentages. Continuous variables were presented as means and standard errors.

Bivariate and multivariable analyses were performed using modified Poisson regression to assess the association between severity of the DFU and the factors studied, with significance determined at p < 0.05. Variables with a p-value < 0.2 in bivariate analysis were considered for multivariable analysis.

Poisson regression was chosen to obtain prevalence risk ratios (PRR) over logistic regression to avoid overestimation of the prevalence ratio and to allow for appropriate control of confounding variables, because the latter poorly estimates the standard errors of the estimated risk ratios especially when dealing with severe DFU which was a common outcome of interest among patients with DFU [39].

### Ethical considerations

Ethical approval was granted by the Research Ethics Committee of Kampala International University (KIU), reference KIU-REC-2021-57 and permission to access the patients was obtained from management of the selected hospitals before data collection.

## Results

Among 122 targeted study patients, 117 (96%) patients with DFU had complete data and this was subsequently analyzed. Five (4%) patients that had incomplete data were excluded from further analysis.

Among the 117 patients with DFU who formed the definitive sample, the majority (n = 70; 59.8%) had severe DFU and 47 (40.2%) less severe DFU. The majority of the patients (61.5%, n = 72) were female. The mean age of study patients was 57.1 years (49.5 for patients with less severe DFU and 67.5 for patients with severe DFU). The majority of the patients (76.9%; n = 90) had poor blood sugar control. Most patients (44%; n = 52) had a grade 3 diabetic foot ulcer followed by grade 2 (28%, n = 33), grade 1 (12%, n = 14), grade 4 (8%, n = 9) and grade 5(8%, n = 9) according to the Wagner classification.

## Severity of the diabetic foot ulcer

**Fig. 2:**
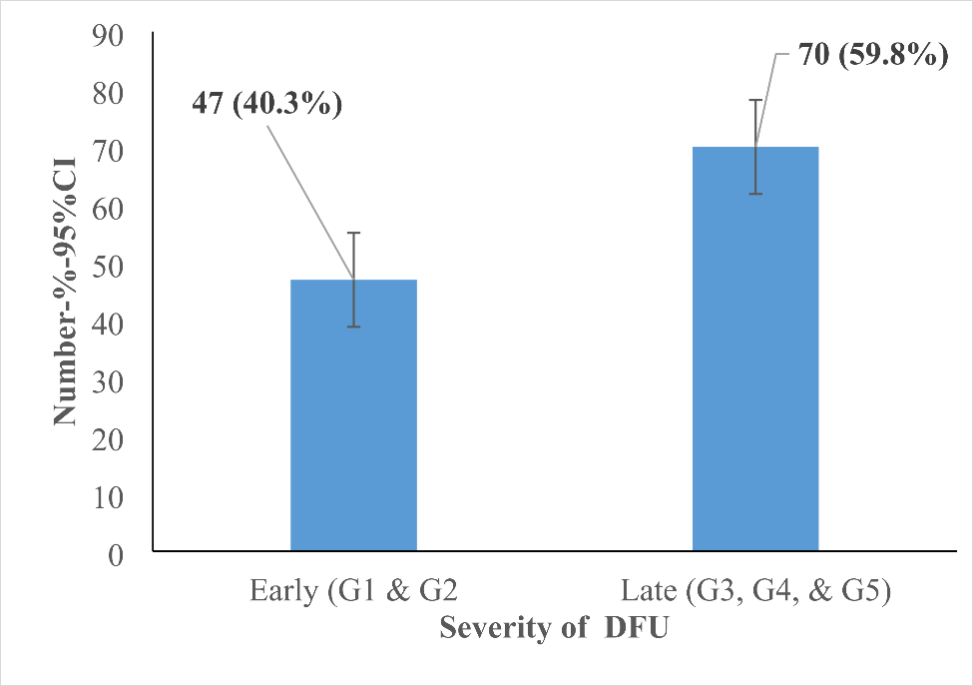
Proportion of patients with severe and less severe diabetic foot ulcers in selected referral hospitals in Uganda, November 2021 to January 2022 (n=117)

As shown in Figs. 2 and 70 (59.8%) of the study patients had severe DFU (95%CI 50.4–68.8) and 47 (40.2%) had less severe DFU (95%CI 31.22–49.6). The proportions were significantly different (p < 0.05).

## Socio demographic characteristics of DFU patients

Most (33.3%) of the patients were in the age-group between 50 and 59 years; and the majority (74.4%) were from rural areas (Table 1). The age group of 70–95 years was associated with severe DFU (p = 0.02).

**Table 1:** Sociodemographic characteristics of patients with DFU in selected referral hospitals in Uganda, November 2021 to January 2022 (n = 117)

Variables	Less severe DFU (G1&G2): n(%)	Severe DFU (G3, G4, &G5): n(%)	Total n (%)	P value
**Age group in years**				0.026
18–39	10 (21.3)	4 (5.7)	14 (12.0)	
40–49	7 (14.9)	8 (11.4)	15 (12.8)	
50–59	13 (27.7)	26 (37.1)	39 (33.3)	
60–69	12 (25.5)	13 (18.6)	25 (21.4)	
70–95	5 (10.6)	19 (27.1)	24 (20.5)	
**Sex**				0.720
Female	28 (38.9)	44 (61.1)	72 (61.5)	
Male	19 (40.4)	26 (37.1)	45 (38.5)	
**Residence**				0.682
Urban	13 (27.7)	17 (24.3)	30 (25.6)	
Rural	34 (72.3)	53 (75.7)	87 (74.4)	
**Region**				0.555
Western	33 (70.2)	51 (72.9)	84 (71.8)	
Central	6 (12.8)	12 (17.1)	18 (15.4)	
Eastern	7 (14.9)	5 (7.1)	12 (10.3)	
Non-Ugandan	1 (2.1)	2 (2.9)	3 (2.6)	
**Occupation**				0.502
Peasant farmer	28 (59.6)	42 (60.0)	70 (59.8)	
Business/self employed	11 (23.4)	13 (18.6)	24 (20.5)	
Non-employed	7 (14.9)	9 (12.9)	16 (13.7)	
Formerly employed	1 (2.1)	6 (8.6)	7 (6.0)	
**Average monthly income in UgShs**				0.144
< 10,000	15 (31.9)	21 (30.0)	36 (30.8)	
10,000-100,000	16 (34.0)	14 (20.0)	30 (25.6)	
100,001-500,000	14 (29.8)	22 (31.4)	36 (30.8)	
500,001-1million	1 (2.1)	9 (12.9)	10 (8.5)	
Above 1million	1 (2.1)	4 (5.7)	5 (4.3)	

Ug.Shs: Ugandan Shillings; 1 United States (US) dollar = 3800Ug.Shs (exchange rate as of 1st September 2022)

## Medical characteristics of DFU patients

Table 2 shows that the majority of patients had DM type 2 (94.9%, n = 111) and 65 (55.6%) had suffered with DM for a duration of 9 years and above. In 55% the DFU had lasted for a duration of 1–6 months, 25.6% had a prior history of DFU and 18.8% had a history of amputation. The majority of patients (65%) were on insulin.

**Table 2:** Medical characteristics of patients in selected referral hospitals in Uganda, November 2021 to January 2022 (n = 117)

Variables	Less severe (G1&G2): n(%)	Severe (G3, G4, &G5): n(%)	Total n (%)	p-value
**Type of DM**				0.174
Type 1	4 (8.5)	2 (2.9)	6 (5.1)	
Type 2	43 (91.5)	68 (97.1)	111 (94.9)	
**Duration of DM in years, mean (SD)**	12.2 (11.0)	10.0 (8.8)	10.9 (9.7)	0.236
**Duration of DM in years**				0.744
0–3	12 (25.5)	19 (27.1)	31 (26.5)	
4–8	10 (21.3)	11 (15.7)	21 (17.9)	
9 and above	25 (53.2)	40 (57.1)	65 (55.6)	
Method of treatment				
**Insulin**				0.834
No	17 (36.2)	24 (34.3)	41 (35.0)	
Yes	30 (63.8)	46 (65.7)	76 (65.0)	
**Herbal medicine**				0.330
No	26 (55.3)	45 (64.3)	71 (60.7)	
Yes	21 (44.7)	25 (35.7)	46 (39.3)	
**Oral hypoglycemic agents (OHA)**				0.783
No	20 (42.6)	28 (40.0)	48 (41.0)	
Yes	27 (57.4)	42 (60.0)	69 (59.0)	
**On treatment**				0.683
No	45 (95.7)	68 (97.1)	113 (96.6)	
Yes	2 (4.3)	2 (2.9)	4 (3.4)	
**Number of types of medication received**				0.173
0	2 (4.3)	2 (2.9)	4 (3.4)	
1	20 (42.6)	39 (55.7)	59 (50.4)	
2	17 (36.2)	13 (18.6)	30 (25.6)	
3	8 (17.0)	16 (22.9)	24 (20.5)	
**Duration of DFU in months, mean (SD)**	4.9 (10.1)	4.2 (7.3)	4.5 (8.5)	0.670
**Duration of DFU in months**				0.527
Less than 1	17 (38.6)	19 (28.4)	36 (32.4)	
1–6	22 (50.0)	39 (58.2)	61 (55.0)	
Over 6	5 (11.4)	9 (13.4)	14 (12.6)	
**History of trauma to an affected foot**				0.604
No	40 (85.1)	57 (81.4)	97 (82.9)	
Yes	7 (14.9)	13 (18.6)	20 (17.1)	
**History of previous DFU**				0.535
No	36 (76.6)	50 (71.4)	86 (73.5)	
Yes	11 (23.4)	20 (28.6)	31 (26.5)	
**History of amputation due to DFU**				0.375
No	40 (85.1)	55 (78.6)	95 (81.2)	
Yes	7 (14.9)	15 (21.4)	22 (18.8)	

SE: standard error

## Behavioral characteristics of patients with DFU

As shown in Table 3, only 20 (17.1%, n = 117) of patients attended a diabetes clinic twice a month and only 22 (18.8%) patients reported to be doing regular physical exercise. The prevalence of other factors associated with severity on DFU such as foot care, history of smoking, not doing physical exercise and eating a mixture of foods are also shown.

**Table 3:** Behavioral characteristics of DFU patients in selected referral hospitals in Uganda, November 2021 to January 2022 (n = 117)

Variables	Early (G1&G2):n(%)	Late (G3, G4, &G5): n (%)	Total n (%)	p-value
**Taught About complications of DM**				0.274
No	20 (42.6)	37 (52.9)	57 (48.7)	
Yes	27 (57.4)	33 (47.1)	60 (51.3)	
**Counselled about risk of DFU when you have DM**				0.411
No	14 (29.8)	26 (37.1)	40 (34.2)	
Yes	33 (70.2)	44 (62.9)	77 (65.8)	
**Counselled by a friend**				0.707
No	31 (66.0)	41 (58.6)	72 (61.5)	
Yes	16 (4.3)	29 (51.4)	45 (38.5)	
**Counselled by a healthcare worker**				0.480
No	30 (63.8)	37 (52.9)	67 (57.3)	
Yes	17(6.4)	33 (47.1)	50 (42.7)	
**Counselled by social media**				0.707
No	31 (66.0)	41 (58.6)	72 (61.5)	
Yes	16 (34.1)	29 (41.4)	45 (39.0)	
**Frequency of DM clinic attendance**				0.577
Never	13 (27.7)	25 (35.7)	38 (32.5)	
Once a month	22 (46.8)	28 (40.0)	50 (42.7)	
Every 2 months	7 (14.9)	13 (18.6)	20 (17.1)	
Once yearly	5 (10.6)	4 (5.7)	9 (7.7)	
**Smoking status**				0.040
Non smoker	44 (93.6)	56 (80.0)	100 (85.5)	
Ever smoked	3 (6.4)	14 (20.0)	17 (14.5)	
**Alcohol consumer**				0.512
No	38 (80.9)	53 (75.7)	91 (77.8)	
Yes	9 (19.1)	17 (24.3)	26 (22.2)	
**Foot care score**				0.464
1	12 (25.5)	25 (35.7)	37 (31.6)	
2	5 (10.6)	5 (7.1)	10 (8.5)	
3	30 (63.8)	40 (57.1)	70 (59.8)	
**Type of shoes worn**				0.908
Open shoes	30 (63.8)	47 (67.1)	77 (65.8)	
Any type of shoes	12 (25.5)	17 (24.3)	29 (24.8)	
Don’t wear shoes	5 (10.6)	6 (8.6)	11 (9.4)	
**How often nails are cut since DM diagnosed**				0.791
Always	14 (29.8)	22 (31.4)	36 (30.8)	
Occasionally	32 (68.1)	45 (64.3)	77 (65.8)	
Don’t	1 (2.1)	3 (4.3)	4 (3.4)	
**Individual who cuts the toenails**				0.762
Self	30 (63.8)	46 (65.7)	76 (65.0)	
Other people	17 (36.1)	24 (34.3)	41 (35.0)	
What do you use to cut your nails (multiple answers)				
**Nail cutter**				0.329
No	33 (70.2)	43 (61.4)	76 (65.0)	
Yes	14 (29.8)	27 (38.6)	41 (35.0)	
**Razor blade**				0.152
No	14 (29.8)	30 (42.9)	44 (37.6)	
Yes	33 (70.2)	40 (57.1)	73 (62.4)	
**Knife**				0.807
No	46 (97.9)	68 (97.1)	114 (97.4)	
Yes	1 (2.1)	2 (2.9)	3 (2.6)	
**Frequency of exercise**				0.529
Always	8 (17.0)	14 (20.0)	22 (18.8)	
Occasionally	27 (57.4)	39 (55.7)	66 (56.4)	
Never	12 (25.5)	17 (24.3)	29 (24.8)	
**Work in the garden**				0.921
No	27 (57.4)	37 (52.9)	64 (54.7)	
Yes	20 (42.6)	33 (47.1)	53 (45.3)	
**Walk at least 30 min a day**				0.625
No	25 (53.2)	47 (67.1)	72 (61.5)	
Yes	22 (46.8)	23 (32.9)	45 (38.5)	
**Housework**				0.128
No	38 (80.9)	56 (80.0)	94 (80.3)	
Yes	9 (19.1)	14 (20.0)	23 (19.7)	
**Jogging**				0.910
No	40 (85.1)	67 (95.7)	107 (91.5)	
Yes	7 (14.9)	3 (4.3)	10 (8.5)	
**No exercise**				0.044
No	36 (76.6)	57 (81.4)	93 (79.5)	
Yes	11 (23.4)	13 (18.6)	24 (20.5)	
**Diet (how often food eaten:)**				
**Vegetables**				0.103
Daily	26(0.2)	32(0.3)	58(0.5)	
Usually	4(0.03)	1(0.01)	5(0.04)	
Occasionally	2(0.02)	8(0.1)	10(0.1)	
Rarely	15(0.1)	29(0.2)	44(0.4)	
**Fruits**				0.924
Daily	21(0.2)	30(0.3)	51(0.4)	
Usually	1(0.01)	1(0.01)	2(0.02)	
Occasionally	6(0.1)	7(0.1)	13(0.1)	
Rarely	19(0.2)	32(0.3)	51(0.4)	
**Fatty food**				0.492
Daily	26(0.2)	39(0.3)	65(0.6)	
Usually	1(0.01)	6(0.1)	7(0.1)	
Occasionally	9(0.1)	10(0.1)	19(0.2)	
Rarely	11(0.1)	15(0.1)	26(0.2)	
**Proteins**				0.215
Daily	30(0.3)	47(0.4)	77(0.7)	
Usually	1(0.01)	6(0.1)	7(0.1)	
Occasionally	8(0.1)	5(0.04)	13(0.1)	
Rarely	8(0.1)	12(0.1)	20(0.2)	
**Carbohydrates**				0.209
Daily	33(0.3)	56(0.5)	89(0.8)	
Usually	4(0.03)	3(0.03)	7(0.1)	
Occasionally	4(0.03)	1(0.01)	5(0.04)	
Rarely	6(0.1)	10(0.1)	16(0.1)	

## Comorbidities of patients with DFU

The study showed that most patients (47.9%) had a normal BMI whilst 38 (32.5%) had pre-obesity. Hypertension was found in 69 (59%) patients, moderate kidney failure in 23 (19.7%), moderate neuropathy in 44 (37.6%) and severe neuropathy in 38 (32.5%) of patients with severe DFU (p-value 0.01). Hba1C levels above 6.5% were found in 90 (76.9%) of patients, compensated heart disease in 22 (18.8%) and decompensated heart disease in 5 (4.3%). Furthermore, claudication was the most common peripheral vascular disease, reported by 29 (24.8%) patients, followed by gangrene (6.0%) and deep venous thrombosis (DVT) (4.3%) (Table 4).

**Table 4:** Co-morbidities of patients with DFU in selected referral hospitals in Uganda, November 2021 to January 2022 (n = 117)

Variables	Early (G1&G2) n(%)	Late (G3, G4, &G5)n(%)	Total n (%)	p-value
**Neuropathy**				0.013
Absent	16 (34.0)	8 (11.4)	24 (20.5)	
Mild	3 (6.4)	8 (11.4)	11 (9.4)	
Moderate	18 (38.3)	26 (37.1)	44 (37.6)	
Severe	10 (21.3)	28 (40.0)	38 (32.5)	
**Peripheral Vascular Disease**				0.103
No disease	35 (74.5)	41 (58.6)	76 (65.0)	
Claudication	10 (21.3)	24 (27.1)	34 (29.1)	
Gangrene	0 (0.0)	7 (10.0)	7 (6.0)	
DVT	2 (4.3)	3 (4.3)	5 (4.3)	
**Hba1C, mean (SD)**	8.3 (2.6)	8.8 (2.6)	8.6 (2.6)	0.158
**Hba1C levels**				
Less than 6.5%	14 (29.8)	13 (18.6)	27 (23.1)	
6.5% and above	33 (70.2)	57 (81.4)	90 (76.9)	0.158
**BP class**				
Normal	19 (40.4)	28 (40.0)	47 (40.2)	
Hypertension	28 (59.6)	41 (58.6)	69 (59.0)	
Hypotension	0 (0.0)	1 (1.4)	1 (0.9)	
**BMI, mean (SD)**	25.9 (6.7)	25.0 (5.2)	25.3 (5.9)	0.393
**BMI group**				0.614
Underweight < 18.5	3 (6.4)	5 (7.1)	8 (6.8)	
Normal 18.5–24.9	21 (44.7)	35 (50.0)	56 (47.9)	
Pre-obesity 25.0–29.9	16 (34.0)	22 (31.4)	38 (32.5)	
Obesity Class I 30.0–34.9	3 (6.4)	6 (8.6)	9 (7.7)	
Obesity Class II 35.0–39.9	2 (4.3)	0 (0.0)	2 (1.7)	
Obesity Class III 40 and above	2 (4.3)	2 (2.9)	4 (3.4)	
**ABI group**				0.192
Normal (1.0 to 1.4)	9(0.1)	19(0.2)	28(0.2)	
Acceptable (0.9 to less 1.0)	4(0.03)	3(0.03)	7(0.1)	
Some Arterial Disease (0.8 to less 0.9)	1(0.01)	0(0.0)	1(0.01)	
Moderate (0.5 to 0.7)	0(0.0)	2(0.02)	2(0.02)	
Severe (Less than 0.5)	0(0.0)	2(0.02)	2(0.02)	
Not assessed	31(0.3)	46(0.4)	77(0.7)	

ABI: ankle brachial index; BMI: body mass index, Hba1C: glycosylated hemoglobin, DVT: deep venous thrombosis, BP: blood pressure, SD = standard deviation

## Anatomical distribution of DFU

Most patients (47.9%, n = 56) had DFU of the right foot, with the majority (54.7%) found on the dorsum of the foot. Location of the ulcer on the plantar region (p = 0.01), having more than 4 ulcers (p = 0.01) and a size of ulcer of > 10 cm of diameter (p = 0.00) were associated with higher severity of the DFU (Table 5).

**Table 5:** Anatomical distribution of DFU among patients in selected referral hospitals in Uganda, November 2021 to January 2022 (n = 117)

Variables	Less severe (G1&G2): n (%)	Severe (G3, G4, &G5): n (%)	Total n(%)	p-value
**Foot affected by DFU**				0.633
Right	23 (48.9)	33 (47.1)	56 (47.9)	
Left	20 (42.6)	27 (38.6)	47 (40.2)	
Both	4 (8.5)	10 (14.3)	14 (12.0)	
**Location of DFU**				
**Heel**				0.409
No	38 (80.9)	52 (74.3)	90 (76.9)	
Yes	9 (19.1)	18 (25.7)	27 (23.1)	
**Dorsum**				0.074
No	26 (55.3)	27 (38.6)	53 (45.3)	
Yes	21 (44.7)	43 (61.4)	64 (54.7)	
**Plantar**				0.009
No	33 (70.2)	32 (45.7)	65 (55.6)	
Yes	14 (29.8)	38 (54.3)	52 (44.4)	
**Toes**				0.788
No	22 (46.8)	31 (44.3)	53 (45.3)	
Yes	25 (53.2)	39 (55.7)	64 (54.7)	
**Number of ulcers**				0.010
1	29 (61.7)	30 (42.9)	59 (50.4)	
2	14 (29.8)	15 (21.4)	29 (24.8)	
3	4 (8.5)	22 (31.4)	26 (22.2)	
4	0 (0.0)	3 (4.3)	3 (2.6)	
**Size of DFU in cm**				< 0.001
1 to 5	34 (72.3)	22 (31.4)	56 (47.9)	
5 to 10	13 (27.7)	29 (41.4)	42 (35.9)	
Over 10	0 (0.0)	19 (27.1)	19 (16.2)	

cm: centimeter

## Factors influencing severity of DFU

The patients with mild neuropathy (aPRR = 3.4; 95% CI = 1.51–7.63; p-value = 0.003) and moderate neuropathy (aPRR = 2.65; 95% CI = 1.34–5.23; p-value = 0.005) were more at risk of developing severe DFU as compared to those with normal foot sensation, when other factors were held constant. Although at bivariate analysis patients with severe neuropathy were twice more likely (95% CI = 1.21–4.03, p = 0.009) to develop severe DFU compared to those with normal foot sensations, this turned out not to be a significant risk factor at multivariable analysis. The age group of 70–95 years was significantly associated with increased risk of severe DFU (APR = 3.02; 95% CI = 1.28–7.17; p = 0.02); the size of the ulcer of more than 5 cm (APR = 1.51; 95%CI= (1.01–2.27); p < 0.05) and size > 10 cm (APR = 2.46;95%=1.38–4.39; p = 0.002). Regular eating of vegetables (APRR = 0.062; 95% CI = 0.004–0.863; p-value = 0.038), education level: primary (APRR = 0.58; 95% CI = 0.38–0.89; p = 0.011), secondary (APR = 0.53; 95% CI = 0.29–0.97; p = 0.039); moderate (APRR = 0.48; 95% CI = ; 95% CI = 0.29–0.78; p = 0.003) and severe visual loss (APR = 0.31; 95%=0.17–0.54; p = 0.011), for 2 ulcers on one foot (APR = 0.52; 95%CI = 0.32–0.86; p = 0.011) were significantly less likely associated with severe DFU (Table 6).

**Table 6:** Bivariate and multivariable analysis for factors associated with severity of DFU in selected referral hospitals in Uganda, November 2021 to January 2022 ( n= 117)

Variable	DFU		Bivariate analysis		Multivariable analysis	
	Less severe n (%)	Severe n (%)	c.PRR (95%CI)	p-value	a.PRR (95%CI)	p-value
**Age**	47 (40.2)	70 (59.8)	1.01(1.01–1.02)	0.003	1.02(1.01–1.03)	0.001
**Age group in years**						
18–39	10 (21.3)	4 (5.7)	1		1	
40–49	7 (14.9)	8 (11.4)	1.87(0.72–4.87)	0.202	2.5(0.91–6.91)	0.076
50–59	13 (27.7)	26 (37.1)	2.33 (0.99–5.52)	0.054	1.43(0.63–3.26)	0.397
60–69	12 (25.5)	13 (18.6)	1.82 (0.73–4.54)	0.199	1.67 (0.72–3.91)	0.234
70–95	5 (10.6)	19 (27.1)	2.77 (1.18–6.53)	0.020	3.023 (1.28–7.17)	0.012
**Sex**						
Female	28 (59.6)	44 (62.9)	1		1	
Male	19 (40.4)	26 (37.1)	0.95 (0.69–1.29)	0.724	-	-
**Residence**						
Rural	34 (72.3)	53 (75.7)	1		1	
Urban	13 (27.7)	17 (24.3)	0.95 (0.69–1.29)	0.724	-	-
**Profession**						
Peasant farmer	28 (59.6)	42 (60.0)	1		1	
Business/self employed	11 (23.4)	13 (18.6)	0.9 (0.6–1.37)	0.630	0.84 (0.43–1.66)	0.621
Non employed	7 (14.9)	9 (12.9)	0.94 (0.58–1.51)	0.790	1.02 (0.61–1.71)	0.939
Formerly employed	1 (2.1)	6 (8.6)	1.43 (1–2.05)	0.052	1.13 (0.5–2.53)	0.773
**Level of education**						
No formal	3 (6.4)	12 (17.1)	1		1	
Primary	29 (61.7)	40 (57.1)	0.73 (0.52–1)	0.052	0.58 (0.38–0.89)	0.011
Secondary	14 (29.8)	14 (20.0)	0.63 (0.4–0.98)	0.041	0.53 (0.29–0.97)	0.039
Tertiary	1 (2.1)	4 (5.7)	1 (0.6–1.66)	1.000	0.45 (0.18–1.17)	0.102
**Religion**						
Muslim	4 (8.5)	9 (12.9)	1		1	
Christian	43 (91.5)	61 (87.1)	0.8 (0.52–1.38)	0.506	0.9 (0.54–1.45)	0.629
**Average monthly income in UgShs**						
< 10,000	15 (31.9)	21 (30.0)	0.73 (0.43–1.23)	0.234	0.42 (0.13–1.33)	0.142
10,000-100,000	16 (34.0)	14 (20.0)	0.58 (0.33–1.05)	0.071	0.39 (0.12–1.31)	0.128
100,001-500,000	14 (29.8)	22 (31.4)	0.76 (0.46–1.28)	0.303	0.47 (0.16–1.42)	0.181
500,001–1,000,000	1 (2.1)	9 (12.9)	1.13 (0.69–1.83)	0.635	0.8 (0.3–2.14)	0.650
Above 1 M	1 (2.1)	4 (5.7)	1	.	1	.
**Smoking status**						
Non smoker	44 (93.6)	56 (80.0)	1	.	1	.
Ever smoked	3 (6.4)	14 (20.0)	1.47 (1.11–1.95)	0.007	1.08 (0.68–1.71)	0.751
**Vegetables**						
Rarely	15(0.1)	29(0.2)	1	.	1	.
Daily	26(0.2)	32(0.3)	0.69 (0.20–2.37)	0.558	4.50 (0.68–1.68)	0.118
Usually	4(0.03)	1(0.01)	0.13 (0.01–1.26)	0.078	0.06 (0.00-0.86)	0.038
Occasionally	2(0.02)	8(0.1)	0.85 (0.38–1.88)	0.685	0.69 (0.28–3.06)	0.430
**Carbohydrates**						
Rarely	6(0.1)	10(0.1)	1	.	1	.
Daily	33(0.3)	56(0.5)	0.15 (0.01–1.68)	0.123	0.18 (0.01–2.40)	0.193
Usually	4(0.03)	3(0.03)	0.45 (0.07–2.74)	0.386	1.08 (0.11–10.30)	0.946
Occasionally	4(0.03)	1(0.01)	1.02 (0.34–3.06)	0.974	1.43 (0.40–5.09)	0.581
**Dorsum**						
No	26 (55.3)	27 (38.6)	1	.	1	.
Yes	21 (44.7)	43 (61.4)	1.32 (0.96–1.81)	0.086	1.32 (0.88–1.98)	0.176
**Plantar**						
No	33 (70.2)	32 (45.7)	1	.	1	.
Yes	14 (29.8)	38 (54.3)	1.48 (1.1–2)	0.009	1.46 (0.89–2.38)	0.135
**Number of locations for one Ulcer**						
1	29 (61.7)	30 (42.9)	1	.	1	.
2	14 (29.8)	15 (21.4)	1.02 (0.66–1.57)	0.938	0.52 (0.32–0.86)	0.011
3	4 (8.5)	22 (31.4)	1.66 (1.23–2.25)	0.001	0.57 (0.27–1.21)	0.145
4	0 (0.0)	3 (4.3)	1.97 (1.53–2.53)	< 0.001	0.72 (0.33–1.6)	0.421
**Visual loss**						
Normal	8 (17.0)	18 (25.7)	1	.	1	.
Mild	7 (14.9)	14 (20.0)	0.96 (0.65–1.43)	0.853	0.55 (0.28–1.08)	0.084
Moderate	15 (31.9)	23 (32.9)	0.87 (0.61–1.26)	0.470	0.48 (0.29–0.78)	0.003
Severe	17 (36.2)	15 (21.4)	0.68 (0.43–1.06)	0.090	0.31 (0.17–0.54)	< 0.001
**Neuropathy**						
Normal	16 (34.0)	8 (11.4)	1	.	1	.
Mild	3 (6.4)	8 (11.4)	2.18 (1.11–4.28)	0.023	3.4 (1.51–7.63)	0.003
Moderate	18 (38.3)	26 (37.1)	1.77 (0.95–3.29)	0.070	2.65 (1.34–5.23)	0.005
Severe	10 (21.3)	28 (40.0)	2.21 (1.21–4.03)	0.009	2.06 (0.96–4.43)	0.063
**Peripheral Vascular Disease**						
No disease	35 (74.5)	41 (58.6)	1	.	1	.
Claudication	10 (21.3)	19 (27.1)	1.21 (0.87–1.7)	0.259	1.39 (0.88–2.2)	0.162
Gangrene	0 (0.0)	7 (10.0)	1.85 (1.51–2.28)	< 0.001	1.26 (0.71–2.24)	0.424
DVT	2 (4.3)	3 (4.3)	1.11 (0.53–2.35)	0.781	1.74 (0.44–6.89)	0.433
Hba1C 6.5%, n (%)						
Less than 6.5%	14 (29.8)	13 (18.6)	1	.		
6.5 and above	33 (70.2)	57 (81.4)	1.32 (0.86–2.01)	0.205	-	-
**Blood Pressure class**						
Normal	19 (40.4)	28 (40.0)	1	.	1	.
Hypertension	28 (59.6)	41 (58.6)	1 (0.73–1.36)	0.987	1.05 (0.64–1.73)	0.851
Hypotension	0 (0.0)	1 (1.4)	1.68 (1.33–2.13)	< 0.001	1.93 (0.56–6.64)	0.296
**Size of DFU in cm**						
1 to 5	34 (72.3)	22 (31.4)	1	.	1	.
5 to 10	13 (27.7)	29 (41.4)	1.76 (1.2–2.58)	0.004	1.51 (1.01–2.27)	0.047
Over 10	0 (0.0)	19 (27.1)	2.55 (1.84–3.53)	< 0.001	2.46 (1.38–4.39)	0.002

UgShs: Ugandan shillings; 1US dollar ($) = 3800ugShs (01, September, 2022); M: million; DVT: Deep venous thrombosis, CPPR: crude prevalence risk ratio, APPR: adjusted prevalence risk ratio

## Discussion

This study assessed the anatomical distribution and factors associated with severity of DFU among patients with DM in Uganda. The majority of patients in our study had poor blood sugar control. The most common location of DFU was the plantar region of the foot unlike other studies which reported metatarsal as the common region [22, 23]. Factors associated with severity of DFU were size of the ulcer, neuropathy and age.

The burden of DM and DFU is increasing worldwide, especially in developing countries such as Uganda [8]. Adult patients have a 10–15% risk of developing DFU during their diabetic lifetime [1, 40]. Our study showed the mean age in years was 57.5 (SD15.2), and older age was significantly associated with higher severity of DFU, especially the 70–95 age group. This result is similar to what was reported in a systematic review where age was associated with severity of DFU [9].

Although our study had a female majority, sex was not significantly associated with severity of DFU. A similar result was reported by Agwu [39] and other authors in Uganda [39, 41]. However, a male majority and preponderance have been reported in other studies [2, 42]. Jupiter [40] in a worldwide systematic and meta-analysis review in found that male patients were significantly more affected by severe DFU than female patients. In addition, level of education, profession, level of income, duration of DM/DFU are significantly associated with the severity of the DFU although these factors have been reported to contribute to the genesis of the DFU [17].

History of smoking was not associated with severity of the DFU in this study although it has been reported to be associated with development of DFU. However, other studies have found history of smoking to be associated with severity of DFU [9, 22]. This finding can be explained by the relatively low number of smokers found in this study. High BMI has been reported to be associated with occurrence of DFU and with severe DFU [9]; however, our results did not support this association. This could be explained by the finding that most patients had normal BMI.

The majority of DFU patients in this study had poor blood sugar control, although this did not appear to be a risk factor associated with severe DFU. This result is different from what has reported in a recent systematic review and by Bekele in Ethiopia where uncontrolled glycemia was significantly associated with severe DFU [9, 42]. Although poor blood sugar control was not associated with severity of DFU in this study, there is still a need to stabilize the sugar levels of all DFU patients.

The majority of DFU patients presented with severe DFU, a finding similar to a report by Smith-Strøm in Norway [43]. However, contrary findings have been reported by Jalilian and colleagues in a systematic review where a higher proportion of patients had less severe DFU [9]. Development of clear guidelines on prevention and cure of DFU with evidence-based specifics for our setting could help reduce the burden of complications due to DFU.

Most patients had Wagner classification Grade 3 DFU which is in agreement with results of a study in Sri Lanka [22]. However, studies from Ethiopia and India, have reported Wagner classification Grade 2 DFU to be the most common among diabetic patients [44–46]. This calls for emphasis on education of patients living with DM to seek medical consultation early in case of any foot wound to reduce progression to severe DFU and its complications in our setting.

Living in a rural area, having a long duration of DM of type 2 or having a history of DFU for more than 6 months was not associated with severity of DFU in this study, although these factors have been reported elsewhere to be associated with development of severe DFU [9, 17, 22].

Our results showed that DFU among patients was mostly on the right foot which concurs with the results of Smith-Strøm H in Norway [43]. Most patients in this study had DFU measuring less than 10cm^2^ which was similar to a study in India where DFU measuring < 10cm^2^was the most common [47].

Many factors have been reported to be associated with the severity of DFU. In this study, a number of factors were identified to be associated with severe DFU and these included neuropathies and the size of the ulcer. This result is similar to a study in Ethiopia where neuropathies were 4 times more likely in patients with severe DFU [47]. This study showed that the larger the ulcer, the more likely it was to be severe. Therefore, DFU should be treated early and effectively to avoid progression towards severity. Ambageda in Sri Lanka did not find any significant association between any factor and severity of DFU [22]. Several studies have reported factors associated with severity of DFU among DM patients [22, 28, 41]. A study in Norway found the duration of the ulcer to be associated with the severity whilst a systematic review by Campbell reported that high HbA1c b was associated with increased severity of DFU [22, 28, 41] and in a systematic review by Jalalian, the location of DFU on the plantar region was associated with increased severity of DFU [9]. Since factors affecting the severity of DFU seem to vary from region to region, location-specific research is recommended in order to contextualize the mitigation of complications of DFU.

## Limitations and strength of this study

The study was hospital-based and this limits the generalization of the results to the entire Ugandan population. Patients interviewed may withhold some information but, in this study, they were assured of confidentiality to avoid this bias. The other limitation is the nature of being a cross-sectional study, where the causal relationship between the contributing factors and the severity of DFU could not be established. Assessment of the vascular insufficiency should have been done using a Doppler scan or CT angiogram which are the recommended tests, however we used the ABI, which was not performed on all patients because of extensive lesions around the ankle joint in some of these patients. The study did not capture data from northern Ugandan and from all major hospitals of Eastern Uganda due to limitation of funds. The strength of this study lies in its multi-center approach and as data was collected concurrently, there is a possibility of generalizing to the catchment area of the study. However, a nationwide prospective study on severity of the DFU and outcomes in the region is proposed.

More attention should be focused upon treating aggressively the neuropathy to prevent extension of the wound.

## Conclusion

Most patients were from western Uganda and have poor health-seeking behavior leading to late consultation in diabetic clinics when the condition is rather severe. The majority of patients had severe DFU and grade 3 was the most common. The dorsum of the right foot was most affected with ulcers and the majority of patients had poor blood sugar control. Primary and secondary educational level, moderate and severe vision loss and eating frequently vegetables were less likely to be associated with severe DFU. There was an association between age of 70–95 years, neuropathies, ulcer size of more than 5 cm and severity of the DFU. Therefore, there is a need to control blood sugar, effectively treat any neuropathy as early as possible and to focus upon patient education to encourage early consultation and prevent the complications associated with severe the DFU.

## Data Availability

The data used to support the findings of this study are available from the corresponding author upon request.

## References

[1] Yazdanpanah L, Shahbazian H, Nazari I, Arti HR, Ahmadi F, Mohammadianinejad SE, Cheraghian B, Hesam S. Incidence and Risk Factors of Diabetic Foot Ulcer: A Population-Based Diabetic Foot Cohort (ADFC Study)-Two-Year Follow-Up Study.Int J Endocrinol. 2018 Mar15;2018:7631659. 10.1155/2018/7631659 PMC5875034 29736169

[2] Jupiter DC, Thorud JC, Buckley CJ, Shibuya N. The impact of foot ulceration and amputation on mortality in diabetic patients. I: from ulceration to death, a systematic review. Int Wound J. 2016 Oct;13(5):892–903. 10.1111/iwj.12404 PMC7950078 25601358

[3] Jeyaraman K, Berhane T, Hamilton M, Chandra AP, Falhammar H. Mortality in patients with diabetic foot ulcer: a retrospective study of 513 cases from a single Centre in the Northern Territory of Australia. BMC EndocrDisord. 2019 Jan 3;19(1):1. 10.1186/s12902-018-0327-2 PMC6318899 30606164

[4] Armstrong DG, Boulton AJM, Bus SA. Diabetic Foot Ulcers and their recurrence. N Engl J Med. 2017 Jun;15(24):2367–75. 10.1056/NEJMra1615439 28614678

[5] Hingorani A, LaMuraglia GM, Henke P, Meissner MH, Loretz L, Zinszer KM, Driver VR, Frykberg R, Carman TL, Marston W, Mills JL, Sr, Murad MH. The management of diabetic foot: a clinical practice guideline by the Society for vascular surgery in collaboration with the american Podiatric Medical Association and the Society for Vascular Medicine. J Vasc Surg. 2016 Feb;63(2 Suppl):3S–21S. 10.1016/j.jvs.2015.10.003 26804367

[6] Rice JB, Desai U, Cummings AK, Birnbaum HG, Skornicki M, Parsons NB (2014). Burden of diabetic foot ulcers for medicare and private insurers. Diabetes Care.

[7] IDF. Eighth edition 2017. 8 TH. Suvi Karuranga, Joao da Rocha Fernandes, Yadi Huang BM, editor. 2017. 150 p.

[8] Driver VR, Fabbi M, Lavery LA, Gibbons G. The costs of diabetic foot: the economic case for the limb salvage team. J Vasc Surg. 2010 Sep;52(3 Suppl):17S–22S. 10.1016/j.jvs.2010.06.003 20804928

[9] Jalilian M, Sarbarzeh PA, Oubari S (2020). Factors related to severity of diabetic foot ulcer: a systematic review. Diabetes Metab Syndr Obes Targets Ther.

[10] Zhang P, Lu J, Jing Y, Tang S, Zhu D, Bi Y. Global epidemiology of diabetic foot ulceration: a systematic review and meta-analysis ^†^. Ann Med. 2017 Mar;49(2):106–16. 10.1080/07853890.2016.1231932 27585063

[11] IDF. (2019). *Idf diabetes atlas* (9TH ed.).

[12] Mariam TG, Alemayehu A, Tesfaye E, Mequannt W, Temesgen K, Yetwale F, Limenih MA (2017). Prevalence of Diabetic Foot Ulcer and Associated factors among adult Diabetic Patients who attend the Diabetic Follow-Up clinic at the University of Gondar Referral Hospital, North West Ethiopia, 2016: institutional-based cross-sectional study. J Diabetes Res.

[13] Bus SA, Lavery LA, Monteiro-Soares M, Rasmussen A, Raspovic A, Sacco ICN, van Netten JJ, International Working Group on the Diabetic Foot. Guidelines on the prevention of foot ulcers in persons with diabetes (IWGDF 2019 update). Diabetes Metab Res Rev. 2020 Mar;36Suppl1:e3269. 10.1002/dmrr.3269 32176451

[14] Amin N, Doupis J. Diabetic foot disease : From the evaluation of the “ foot at risk ” to the novel diabetic ulcer treatment modalities. 2016;7(7):153–64. 10.4239/wjd.v7.i7.153 PMC4824686 27076876

[15] Viswanathan. Pattern and Causes of Amputation in Diabetic Patients – A Multicentric Study from India.J Assoc Physicians India ·. 2011;59(March). 21751622

[16] Deribe (2014). Prevalence and factors influencing Diabetic Foot Ulcer among Diabetic. J Diabetes Metab.

[17] Zulfiqarali (2017). Managing the diabetic foot in resource-poor settings : challenges and solutions. Chronic Wound Care Manag Res.

[18] Reardon R, Simring D, Kim B, Mortensen J, Williams D, Leslie A. The diabetic foot ulcer. Aust J Gen Pract. 2020 May;49(5):250–5. 10.31128/AJGP-11-19-5161 32416652

[19] Seid. Knowledge, Practice, and Barriers of Foot Care among Diabetic Patients Attending Felege Hiwot Referral Hospital, Bahir Dar, Northwest Ethiopia.Hindawi Publ Corp. 2015;2015.

[20] Mehmood MK, Parkar AZ, Nayab TM, Mustafa SS, Makin MA, Alawadi F, Farghaly S. (2019). Diabetic foot self-care: awareness and practice among type 2 diabetic patients in primary healthcare centers, Dubai Health Authority. Int J Community Med Public Heal. 2019;6(1):1–7.

[21] Sari Y, Upoyo AS, Isworo A, Taufik A, Sumeru A, Anandari D, Sutrisna E (2020). Foot self-care behavior and its predictors in diabetic patients in Indonesia. BMC Res Notes.

[22] Ambegoda ALAMC, Wijesekera JR, Panditharathne KI, Gamage RT, Mudalige OM D. C. S., &, Piyasiri MDRM. (2016). Analysis of Severity and Anatomical Distribution of Diabetic Foot Ulcers-A Single Unit Experience. Int J Multidiscip Stud. 2015;2(I):12–21.

[23] Patil A, More D, Patil A, Jadhav KA, Mejia MEV, Patil SS, Clinical. Etiological, Anatomical, and Bacteriological Study of “ Diabetic Foot ”Patients : Results of a Single Center Study. 2018;10(4). 10.7759/cureus.2498 PMC6005397 29928559

[24] Bahendeka S, Wesonga R, Mutungi G, Muwonge J, Neema S, Guwatudde D. Prevalence and correlates of diabetes mellitus in Uganda: a population-based national survey. Trop Med Int Health. 2016 Mar;21(3):405–16. 10.1111/tmi.12663. Epub 2016 Jan 21. PMID: 26729021. 10.1111/tmi.12663 26729021

[25] Singh A, Masuku M (2014). Sampling techniques & determination of sample size in Applied Statistics Research: an overview. Ijecm Co Uk.

[26] Assaad-Khalil SH, Zaki A, Rehim AA, Megallaa MH, Gaber N, Gamal H (2015). Prevalence of diabetic foot disorders and related risk factors among egyptian subjects with diabetes. Prim Care Diabetes [Internet].

[27] Maingi W, Kikuvi G, Matheri J (2020). Prevalence and factors Associated with Diabetic Foot Ulcer among adult patients attending Diabetic Clinic at Nyeri Level 5 Hospita. Afr J Health Sci Volume.

[28] Campbell L, Pepper T, Shipman K. HbA1c: a review of non-glycaemic variables.J Clin Pathol. 2019;12–9. 10.1136/jclinpath-2017-204755 30361394

[29] Melaku T, Wondmagegn H, Gebremickael A, Tadesse A. Patterns of superficial veins in the cubital fossa and its clinical implications among southern Ethiopian population.Anat Cell Biol. 2022 Jun30;55(2):148–154. doi: 10.5115/acb.21.217 10.5115/acb.21.217 PMC9256478 35383135

[30] Shi L, Wei H, Zhang T, Li Z, Chi X, Liu D et al. A potent weighted risk model for evaluating the occurrence and severity of diabetic foot ulcers.Diabetol Metab Syndr [Internet]. 2021;1–11. 10.1186/s13098-021-00711-x PMC8407043 34465375

[31] Nuttall FQ (2015). Body Mass Index Nutr Res.

[32] Bergler-Klein J. What’s new in the ESC 2018 guidelines for arterial hypertension: the ten most important messages. Wien KlinWochenschr. 2019 Apr;131(7–8):180–5. 10.1007/s00508-018-1435-8 PMC6459798 30715608

[33] Drechsel TJ, Monteiro RL, Zippenfennig C, Ferreira JSSP, Milani TL, Sacco ICN (2021). Low and high frequency vibration perception thresholds can improve the diagnosis of Diabetic Neuropathy. J Clin Med.

[34] Lai S, Ahmed U, Bollineni A, Lewis R, City K, Angeles L (2016). HHS Public Access.

[35] Picon AP, Ortega NRS, Watari R, Sartor C, Sacco ICN (2012). Classification of the severity of diabetic neuropathy: a new approach taking uncertainties into account using fuzzy logic. Clinics.

[36] Yu Y. Gold Standard for Diagnosis of DPN. Front Endocrinol (Lausanne). 2021 Oct 26;12:719356. doi: 10.3389/fendo.2021.719356. PMID: 34764937; PMCID: PMC8576350. 10.3389/fendo.2021.719356 PMC8576350 34764937

[37] Dyck PJ, Bushek W, Spring EM, Karnes JL, Litchy WJ, O’Brien PC (1987). Vibratory and cooling detection thresholds compared with other tests in diagnosing and staging diabetic neuropathy. Diabetes Care.

[38] Al Ashwal SM (2021). Prevalence and determinants of visual impairment among School Children in Qatar. Int Arch Public Health Community Med.

[39] Yelland LN, Salter AB, Ryan P. Practice of Epidemiology Performance of the Modified Poisson Regression Approach for Estimating Relative Risks From Clustered Prospective Data. 2011;174(8):984–92. 10.1093/aje/kwr183 21841157

[40] Agwu E, Dafiewhare EO, Ekanem PE. Possible Diabetic-Foot Complications in Sub-Saharan Africa. 2011;(June 2014).

[41] Atwine (2015). Health-care seeking behaviour and the use of traditional medicine among persons with type 2 diabetes in south-western Uganda: a study of focus group interviews. pana Afr Med J.

[42] Bekele F, Chelkeba L, Fekadu G, Bekele K (2020). Risk factors and outcomes of diabetic foot ulcer among diabetes mellitus patients admitted to Nekemte referral hospital, western Ethiopia: prospective observational study. Ann Med Surg [Internet].

[43] Smith-Strøm H, Iversen MM, Igland J, Østbye T, Graue M, Skeie S (2017). Severity and duration of diabetic foot ulcer (DFU) before seeking care as predictors of healing time: a retrospective cohort study. PLoS ONE.

[44] Gebrekirstos. Prevalence and Factors Associated With Diabetic Foot Ulcer among Adult Diabetes & Metabolism Prevalence and Factors Associated With Diabetic Foot Ulcer among Adult Patients in Ayder Referral Hospital Diabetic Clinic Mekelle, North. Diabetes Metab. 2016;(January 2015).

[45] Jeon BJ, Choi HJ, Kang JS, Tak MS, Park ES (2017). Comparison of five systems of classification of diabetic foot ulcers and predictive factors for amputation. Int Wound J.

[46] Zubair M, Malik A, Ahmad J (2011). Clinico-microbiological study and antimicrobial drug resistance profile of diabetic foot infections in North India. Foot.

[47] Asegid R, Befikadu T, Esekezaw A, Busera S (2021). Magnitude of Diabetic Foot Ulcer and Associated factors among Diabetic Patients who attended Diabetic follow-up clinics in Gamo and Gofa Zones, Southern Ethiopia. Int J Diabetes Clin Res.

